# Deciphering *Butea monosperma* Kombucha Fermentation Through NMR and Live‐Cell Imaging

**DOI:** 10.1002/fsn3.72531

**Published:** 2026-11-03

**Authors:** Suman Bharti, Dinesh Chandra Rai, Arvind Kumar, Anand Kumar Pandey, Yada Nolvachai, Dipendra Kumar Mahato

**Affiliations:** ^1^ Department of Dairy Science and Food Technology, Institute of Agricultural Sciences Banaras Hindu University Varanasi India; ^2^ Babasaheb Bhimrao Ambedkar Bihar University Muzaffarpur India; ^3^ Department of Biotechnology Engineering Bundelkhand University Jhansi India; ^4^ CASS Food Research Centre, School of Exercise and Nutrition Sciences Deakin University Burwood Victoria Australia; ^5^ ARC Training Centre for Hyphenated Analytical Separation Technologies (HyTECH) Melbourne Victoria Australia

**Keywords:** fermentation, kombucha, live‐cell imaging, NMR, SCOBY

## Abstract

This study employed live‐cell imaging and nuclear magnetic resonance (NMR) spectroscopy to investigate the microbiological and metabolic changes occurring during the fermentation of *B. monosperma* (Palash) flower kombucha. Throughout the 14‐day fermentation period, live‐cell imaging enabled direct visualization of SCOBY development, showing the progressive formation of a dense and interconnected network of cellular and aggregate structures. Semi‐quantitative ImageJ analysis showed marked increases in image‐derived surface coverage, particle count, and aggregate size, consistent with active fermentation. Complementary ^1^H and ^13^C NMR analyses revealed fermentation‐associated changes in the chemical composition of both green tea kombucha (GTK) and Palash flower kombucha (PFK). These included alterations in carbohydrate‐ and glycoside‐associated resonances and the appearance of additional signals consistent with microbial transformation of tea constituents. Fermentation also produced significant physicochemical and antioxidant changes. The pH decreased from 6.0–6.2 to 2.90 and 2.88, while titratable acidity increased to 1.87% and 1.73% in GTK and PFK, respectively. Total soluble solids decreased from 11.6–11.9 Brix to 5.0 Brix in GTK and 6.5 Brix in PFK. In PFK, DPPH radical‐scavenging activity increased from 60.93% to 80.41%, total phenolic content increased from 128.99 to 263.24 mg GAE/L, and total flavonoid content reached 94.74 mg QE/L. Overall, fermentation improved the physicochemical properties, antioxidant capacity, and chemical composition of Palash flower kombucha, highlighting its potential as a functional fermented beverage.

## Graphical abstract

Green tea (control) and *Butea monosperma* flower infusions were prepared with sugar and a symbiotic culture of bacteria and yeast (SCOBY), followed by 14 days of fermentation. The resulting kombucha samples were evaluated for physicochemical properties and antioxidant activity. Live‐cell imaging was used to visualize microscopic changes during fermentation, while nuclear magnetic resonance (NMR) spectroscopy was employed to compare the chemical profiles of the samples. This integrated approach combines physicochemical, antioxidant, microscopic, and spectroscopic analyses to investigate kombucha fermentation.

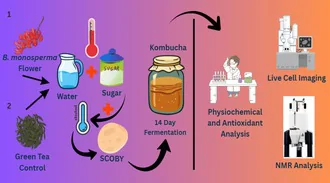

## 1 Introduction

Kombucha is a fermented beverage produced by a symbiotic culture of bacteria and yeast (SCOBY) in sweetened tea. It is gaining popularity because of its anti‐inflammatory, antimicrobial, and antioxidant properties (Anantachoke et al. 2023). The metabolic complexity of kombucha arises from microbial fermentation pathways that transform sugars and tea polyphenols, producing a diverse range of metabolites, including organic acids, vitamins, and other bioactive compounds, while also modifying existing phenolic constituents (Huang 2024). Global demand for kombucha is driven by consumer interest in its functional properties and novel flavors (Alkan et al. 2025). Flavored kombucha accounts for more than 60% of global demand, reflecting a growing preference for functional yet sensory‐enhanced products (Onsun et al. 2025).

To meet this demand, research has increasingly focused on new fermentation substrates, particularly floral and herbal materials (Alkan et al. 2025). The use of *B. monosperma* (Palash) flowers as an alternative substrate offers an opportunity to diversify kombucha formulations and investigate their functional potential. These flowers contain diverse bioactive constituents, including flavonoids and other phenolic compounds, and have a documented history of traditional medicinal use (Garg et al. 2026).

Physicochemical parameters, antioxidant activity, total phenolic content (TPC), and total flavonoid content (TFC) are widely used to evaluate the bioactive properties of kombucha. Fermentation can enhance the availability of phenolic and flavonoid compounds, thereby improving antioxidant potential (Jayabalan et al. 2014; Villarreal‐Soto et al. 2018).

Advanced analytical techniques can be used to study the dynamics of this fermentation. Nuclear magnetic resonance (NMR) spectroscopy enables time‐dependent monitoring of metabolite transformations during fermentation (Forsberg et al. 2024). Beyond changes in chemical composition, fermentation involves spatial organization and microbial interactions. NMR spectroscopy was therefore complemented by live‐cell imaging to provide complementary visual information on the structural development of the fermentation system. Microscopy can reveal the spatial organization of microbial cells and cellulose within kombucha biofilms, providing structural insights that chemical analyses alone cannot capture (Tran et al. 2021).

The present study therefore combines NMR analysis with live‐cell imaging to characterize the biochemical and microbiological changes during kombucha fermentation using *B. monosperma* flowers as a substrate.

## 2 Materials and Methods

The parent culture for fermentation was a stable, mature SCOBY pellicle purchased from Peepal Farm (Amazon, India; batch number 260509). Fresh *B. monosperma* flowers were collected from the botanical garden on the BHU campus, Varanasi, India, and then cleaned and tray‐dried. Yaongyimsen green tea was procured from Mokokchung, Nagaland, India.

### 2.1 Sample Preparation

The sample preparation procedure was adapted from Agatha et al. (2021). Dried *B. monosperma* flowers (8 g/L) were infused in boiled water for 15 min to obtain the floral extract. After infusion, 7% (w/v) sucrose was added to prepare the sweetened flower tea. The tea was inoculated with 10% (v/v) kombucha starter culture containing SCOBY and fermented under static conditions at 25°C for 14 days in sterile 2 L glass vessels. The vessels were lightly covered with breathable cloth to permit gas exchange while minimizing external contamination. Three independent biological replicates were prepared, each in a separate fermentation vessel. After fermentation, the kombucha was filtered to remove the SCOBY biomass. Green tea kombucha, used as the control, was prepared in parallel by the same procedure, using green tea leaves as the substrate.

### 2.2 Experimental Design

The study was conducted using two kombucha substrates: green tea and Palash flower ( *B. monosperma* ). Three independent green tea substrate preparations (GT1, GT2, and GT3) and three independent Palash flower substrate preparations (PFT1, PFT2, and PFT3) were prepared under identical conditions using substrate, sucrose, and water. Samples were collected before SCOBY inoculation and analyzed as unfermented controls.

Each substrate preparation was then inoculated with SCOBY and fermented for 14 days under identical conditions. After fermentation, three independent green tea kombucha samples (GTK1, GTK2, and GTK3) and three independent Palash flower kombucha samples (PFK1, PFK2, and PFK3) were collected for analysis. In total, 12 independent biological samples (six unfermented and six fermented) were evaluated. The effect of fermentation on pH, titratable acidity, TSS, antioxidant activity, TPC, TFC, and NMR profiles was assessed by comparing each unfermented substrate with its corresponding fermented kombucha.

### 2.3 Microscopy Imaging

Bright‐field microscopy was used to descriptively characterize SCOBY‐associated cellular and aggregate structures during kombucha fermentation. Imaging was performed on a Zeiss Celldiscoverer 7 automated high‐content imaging system (Carl Zeiss AG, Germany) equipped with a Plan‐Apochromat 20×/0.95 objective and a 2× Optovar (tube lens). This configuration provided a total magnification of 40× and a calibrated pixel resolution of 0.113 μm/pixel (image size: 1812 × 1306 pixels; field of view: 205.44 × 148.07 μm). Images were acquired in bright‐field contrast mode using a TL LED transmitted‐light lamp at 10% intensity and captured with a 14‐bit camera detector via ZEN 3.2 (blue edition) software (Carl Zeiss AG, Germany). Liquid samples were placed on standard glass microscope slides, covered with coverslips, and mounted in a three‐slot Zeiss slide holder. No dyes or stains were used, so that the native appearance of the samples was preserved.

For microscopy, a single, separate Palash flower kombucha fermentation vessel was monitored longitudinally throughout the 14‐day fermentation period. Images were acquired from this vessel on Days 1, 3, 7, 10, and 14. At each time point, three nonoverlapping microscopic fields suitable for analysis were manually selected and analyzed using ImageJ. These fields were treated as technical subsamples rather than independent biological replicates. Microscopy‐derived measurements are therefore reported as mean ± SD of the three fields at each time point, representing field‐to‐field variation within the same vessel rather than biological variability.

Imaging was performed under fixed acquisition conditions; however, field selection was not randomized or blinded. Because the samples were examined as liquid preparations under a coverslip, variations in liquid depth could not be independently controlled. The microscopy observations and ImageJ‐derived measurements were therefore considered descriptive and exploratory and were not used to infer statistical differences at the biological‐replicate level.

### 2.4 Semi‐Quantitative ImageJ Analysis

All image analyses were conducted using ImageJ version 1.54 within the Fiji distribution (National Institutes of Health, Bethesda, MD, USA) (Schindelin et al. 2012; Schneider et al. 2012). Images were imported without pre‐processing or contrast enhancement to preserve the original intensity information. Although a calibrated pixel resolution (0.113 μm/pixel) was available, the comparative ImageJ analysis used pixel‐based measurements. Measurements were performed on the three microscopic fields per time point described in Section 2.3.

For pellicle coverage analysis, the visible pellicle boundary was manually delineated as a circular region of interest (ROI). Following conversion to 8‐bit grayscale, image segmentation was performed using Otsu automatic thresholding (Otsu 1979). Morphometric descriptors, including pellicle/surface coverage, particle circularity, aspect ratio, and solidity, were extracted using the Analyze Particles function in ImageJ (Schneider et al. 2012). Differential sub‐zone coverage was quantified using dual grayscale threshold segmentation, with dark pigmented regions defined by gray values of 0–100 and translucent/light regions by gray values of 101–200. Radial intensity distributions were generated from the pellicle centroid to the periphery using the Plot Profile function.

Rod‐shaped morphotypes were operationally classified as segmented objects with an aspect ratio > 2.0 and circularity < 0.6; this classification was based on object shape only. Aggregate clusters were defined as connected segmented regions exceeding 500 px^2^ in projected area. Particle/aggregate counts were derived from individual microscopic fields and were not directly equated with pellicle/surface coverage. Collectively, these semi‐quantitative image‐derived parameters were used to descriptively characterize temporal changes in SCOBY‐associated surface coverage, particle/aggregate morphology, and structural heterogeneity. The microscopy data were not used to infer microbial identity, viability, proliferation, or succession.

### 2.5 pH, Titratable Acidity, and TSS Analysis

Changes in pH, titratable acidity (TA), and total soluble solids (TSS) were monitored during fermentation. The pH of the kombucha samples was measured using a calibrated digital pH meter (Lab Man Scientific Instruments, India).

TA was determined by direct acid–base titration following Elfirta et al. (2024), with minor modification. Briefly, 10 mL of undiluted kombucha was titrated with standardized 0.1 *N* NaOH using three drops of 1% (w/v) phenolphthalein as the indicator until a faint pink color persisted for 15 s (approximately pH 8.2). TA was expressed as % acetic acid equivalent and calculated as:

$$\mathrm{TA}\;\left(\%\text{acetic acid}\right)=\frac{V\times N\times 60.05\times 100}{1000\times V_{s}}$$

where *V* is the volume (mL) of NaOH consumed, *N* is the NaOH normality (0.1 N), 60.05 is the equivalent weight of acetic acid, and *V*_s_ is the sample volume (10 mL). Analyses were performed using three independent biological replicates, and results are presented as mean ± SD.

TSS was determined using a digital refractometer (Megabrix/REDI‐P‐101, São Paulo, Brazil) and expressed as Brix.

### 2.6 Antioxidant Activity, TPC, and TFC Analysis

Antioxidant activity was determined using the 2,2‐diphenyl‐1‐picrylhydrazyl (DPPH) radical‐scavenging assay following Brand‐Williams et al. (1995). Total phenolic content (TPC) was quantified using the Folin–Ciocalteu colorimetric method (Singleton and Rossi 1965) and expressed as mg gallic acid equivalents (GAE)/L. Total flavonoid content (TFC) was determined using the aluminum chloride colorimetric assay described by Chang et al. (2002) and expressed as mg quercetin equivalents (QE)/L. Absorbance measurements were recorded using a UV–Visible spectrophotometer at 517, 765, and 415 nm for DPPH, TPC, and TFC, respectively.

### 2.7 NMR Spectroscopic Analysis

Approximately 20 mg of each lyophilized sample (unfermented and fermented *B. monosperma* flower tea and green tea) was dissolved in 600 μL of deuterium oxide (D_2_O, 99.9% D) and transferred to a standard 5 mm NMR tube. Spectra were acquired on a DELTA2 NMR spectrometer equipped with a broadband probe (Department of Chemistry, Banaras Hindu University), operating at 500 MHz for ^1^H and 125.76 MHz for ^13^C. ^1^H spectra were recorded with 32 scans and a relaxation delay of 5 s, and ^13^C spectra with 1024 scans and a relaxation delay of 2 s. Spectra were acquired as standard pulse–acquire experiments without a dedicated water‐suppression pulse sequence. No internal chemical‐shift reference standard (e.g., TSP) was added. Spectra were referenced using the default calibration in MestReNova version 14.2.1, which was also used for baseline correction, phase correction, and peak identification by visual comparison with literature chemical‐shift values. The dominant resonance at *δ* 4.5–4.8 ppm was attributed to residual HDO and excluded from metabolite interpretation because its position varied between samples in a manner characteristic of the residual solvent peak (Gottlieb 1997). Compound assignments were based on comparison with published literature and were not independently confirmed using authentic standards, peak spiking, or two‐dimensional NMR experiments (Sumner et al. 2007).

### 2.8 Statistical Analysis

Data were expressed as mean ± standard deviation (SD). Statistical differences among treatments were evaluated using one‐way analysis of variance (ANOVA) followed by Tukey's honestly significant difference (HSD) post hoc test at a significance level of *p* < 0.05. Pearson's correlation analysis was performed to assess the relationships among antioxidant activity, total phenolic content, and total flavonoid content.

## 3 Results and Discussion

### 3.1 Temporal Microscopy Imaging

Bright‐field microscopy images acquired from Day 1 to Day 14 (Figure 1A) and the corresponding processed images (Figure 1B) were compared descriptively under fixed acquisition conditions. The comparison characterized temporal changes in the appearance, distribution, and organization of visible particle‐ and surface‐associated structures during fermentation.

**FIGURE 1:**
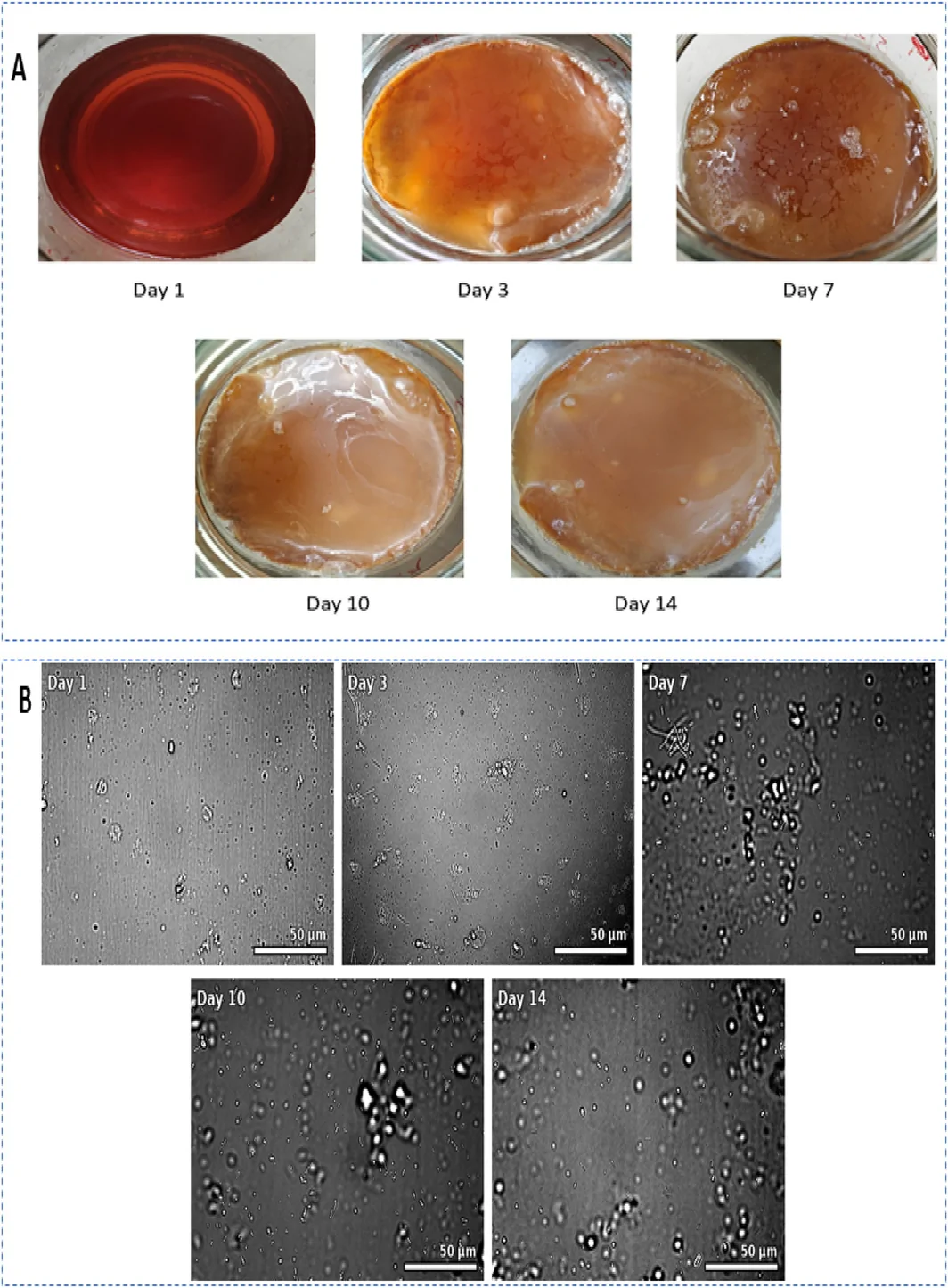
Temporal changes in *B. monosperma* flower kombucha during 14 days of fermentation, shown for one representative fermentation vessel. (A) Macroscopic appearance at Days 1, 3, 7, 10, and 14 under standard laboratory lighting. (B) Corresponding bright‐field photomicrographs from the same fermentation vessel and time points (40× total magnification; scale bar = 50 μm). Images were processed using identical contrast settings across time points for visual comparability only; raw images were used for ImageJ‐based quantitative analysis.

The unfermented control (sweetened *B. monosperma* flower tea without SCOBY inoculation; Day 1 PFT) showed no prominent cellular or aggregate‐associated structures under the imaging conditions used. In contrast, the SCOBY‐inoculated samples showed progressive changes in the abundance and organization of visible structures. These structures were comparatively limited during Days 3–7 and increased markedly at Days 10 and 14; by Day 14, extensive aggregated and surface‐associated structures were evident in the analyzed fields.

These temporal changes coincided with the physicochemical changes during fermentation. In our previous study, the pH of *B. monosperma* flower kombucha decreased from 3.32 on Day 1 to 2.72 on Day 14, indicating progressive acidification (Bharti et al. 2026). Such changes may influence the organization and persistence of surface‐associated structures. However, unstained bright‐field microscopy cannot determine microbial identity, viability, proliferation, or succession. The observations are therefore interpreted only as descriptive changes in visible morphology, particle/aggregate distribution, and surface‐associated structures.

### 3.2 Semi‐Quantitative ImageJ Analysis

Table 1 presents the ImageJ‐derived morphometric measurements from three microscopic fields at each time point. As described in Section 2.3, these fields are technical subsamples from a single fermentation vessel; the data are therefore interpreted descriptively, and the standard deviation represents field‐to‐field variation.

**TABLE 1:** Semi‐quantitative ImageJ‐derived morphometric parameters of SCOBY‐associated structures obtained from bright‐field microscopy during kombucha fermentation at Days 1, 3, 7, 10, and 14.

Parameter (ImageJ)	Day 1	Day 3	Day 7	Day 10	Day 14
Pellicle/surface coverage (%)	0.4 ± 0.1	6.8 ± 0.7	18.4 ± 1.2	31.7 ± 1.8	52.6 ± 2.3
Particle/aggregate count (per frame)	4 ± 1	44 ± 5	127 ± 11	224 ± 18	312 ± 24
Mean gray value (0–255)	193 ± 8	172 ± 9	148 ± 11	118 ± 13	94 ± 10
Texture StdDev	12.4 ± 1.8	18.6 ± 2.1	29.8 ± 3.0	39.4 ± 3.6	47.8 ± 4.1
Mean circularity (0–1)	0.84 ± 0.04	0.79 ± 0.05	0.72 ± 0.05	0.66 ± 0.06	0.61 ± 0.07
Mean particle area (px^2^)	38 ± 6	52 ± 9	118 ± 22	284 ± 48	596 ± 87
Aggregate cluster coverage (%)	0	0.4 ± 0.1	3.2 ± 0.5	9.8 ± 1.1	18.4 ± 1.9
Rod‐shaped morphotype ratio (%)	12 ± 3	18 ± 4	41 ± 6	38 ± 5	22 ± 4

*Note:* Values are presented as mean ± SD of three microscopic fields from the same fermentation vessel at each time point.

Pellicle/surface coverage increased progressively from 0.4% ± 0.1% on Day 1 to 6.8% ± 0.7% on Day 3, 18.4% ± 1.2% on Day 7, 31.7% ± 1.8% on Day 10, and 52.6% ± 2.3% on Day 14. This represents a 16.4‐fold increase in image‐derived surface coverage over the observation period (*R* ^2^ = 0.98). The increase indicates that a greater proportion of each analyzed field was occupied by visible surface‐associated structures. The most pronounced increase occurred between Days 7 and 14, coinciding with the visible development and consolidation of surface‐associated material. These observations are consistent with the reported formation of cellulose‐rich pellicles and aggregated structures during kombucha fermentation (Jayabalan et al. 2014; Villarreal‐Soto et al. 2018).

Particle/aggregate count increased from 4 ± 1 objects per frame on Day 1 to 44 ± 5 on Day 3, 127 ± 11 on Day 7, 224 ± 18 on Day 10, and 312 ± 24 on Day 14. The increase in object number was accompanied by an increase in mean object area from 38 ± 6 px^2^ on Day 1 to 596 ± 87 px^2^ on Day 14, indicating progressively larger segmented structures. Similarly, aggregate cluster coverage increased from 0% on Day 1 to 18.4% ± 1.9% on Day 14, indicating more extensive spatial clustering.

The proportion of rod‐shaped morphotypes increased from 12% ± 3% on Day 1 to 18% ± 4% on Day 3 and 41% ± 6% on Day 7, then decreased to 38% ± 5% on Day 10 and 22% ± 4% on Day 14. Elongated objects were therefore most represented among the segmented structures at Day 7. Because different microorganisms and non‐biological structures can have overlapping shapes under unstained bright‐field microscopy, this parameter was used solely as a morphometric descriptor.

Mean gray value decreased from 193 ± 8 on Day 1 to 94 ± 10 on Day 14, whereas texture standard deviation increased from 12.4 ± 1.8 to 47.8 ± 4.1. The decrease in mean gray value and increase in texture variation indicate changes in the optical characteristics and spatial heterogeneity of the analyzed fields. In parallel, mean circularity decreased from 0.84 ± 0.04 to 0.61 ± 0.07, indicating a shift from relatively regular segmented objects toward more irregular structures. These parameters therefore provide descriptive measures of changes in image‐derived structural complexity during fermentation.

The microscopic fields also differed over time in the apparent morphology of visible structures. Day 3 fields contained relatively more spherical to oval structures, whereas elongated and clustered structures became more prominent at later time points. Such features may occur within the mixed microbial aggregates reported in kombucha; however, morphology alone cannot establish the identity, viability, or functional role of individual microorganisms (Marsh et al. 2014; Villarreal‐Soto et al. 2018).

Overall, image‐derived surface coverage, particle/aggregate count, mean object area, aggregate clustering, and texture variation increased over time, whereas mean gray value and circularity decreased. These measurements indicate increasing structural heterogeneity and organization of visible SCOBY‐associated material during fermentation.

### 3.3 pH, Titratable Acidity, and TSS Analysis

The pH, TA, and TSS of GTK and PFK changed markedly during the 14‐day fermentation, indicating active microbial metabolism (Figure 2). One‐way ANOVA showed significant differences among groups for pH (*F* = 16256.21, *p* < 0.001), titratable acidity (*F* = 434.83, *p* < 0.001), and TSS (*F* = 693.02, *p* < 0.001). The initial pH of both substrates (6.0–6.2) decreased to 2.90 in GTK and 2.88 in PFK, a reduction of approximately 3.1–3.3 pH units. This decline is attributed to the production of organic acids, including acetic, gluconic, and glucuronic acids, by the SCOBY consortium (Villarreal‐Soto et al. 2018; Laavanya et al. 2021; Bharti et al. 2026). Concurrently, titratable acidity increased from approximately 0.10% in the unfermented controls to 1.87% in GTK and 1.73% in PFK, an approximately 17‐ to 19‐fold increase. This increase reflects the conversion of sugars to ethanol by yeasts, followed by the oxidation of ethanol to organic acids by acetic acid bacteria (Chakravorty et al. 2016; Villarreal‐Soto et al. 2018). TSS decreased from 11.6–11.9 Brix to 5.0 Brix in GTK and 6.5 Brix in PFK. This decrease may be partly attributed to microbial utilization of sugars, together with changes in other dissolved components such as organic acids and ethanol. Tukey's HSD test showed no significant difference between GTK and PFK in pH (*p* = 0.913) or titratable acidity (*p* = 0.264), indicating comparable acidification. In contrast, TSS differed significantly between GTK and PFK (Figure 2c). The higher residual TSS in PFK suggests somewhat lower sugar utilization in the *B. monosperma* flower substrate than in green tea under the same conditions, consistent with our previous findings (Bharti et al. 2026). These results confirm successful fermentation of both substrates and highlight the potential of *B. monosperma* flowers as a novel raw material for functional kombucha production.

**FIGURE 2:**
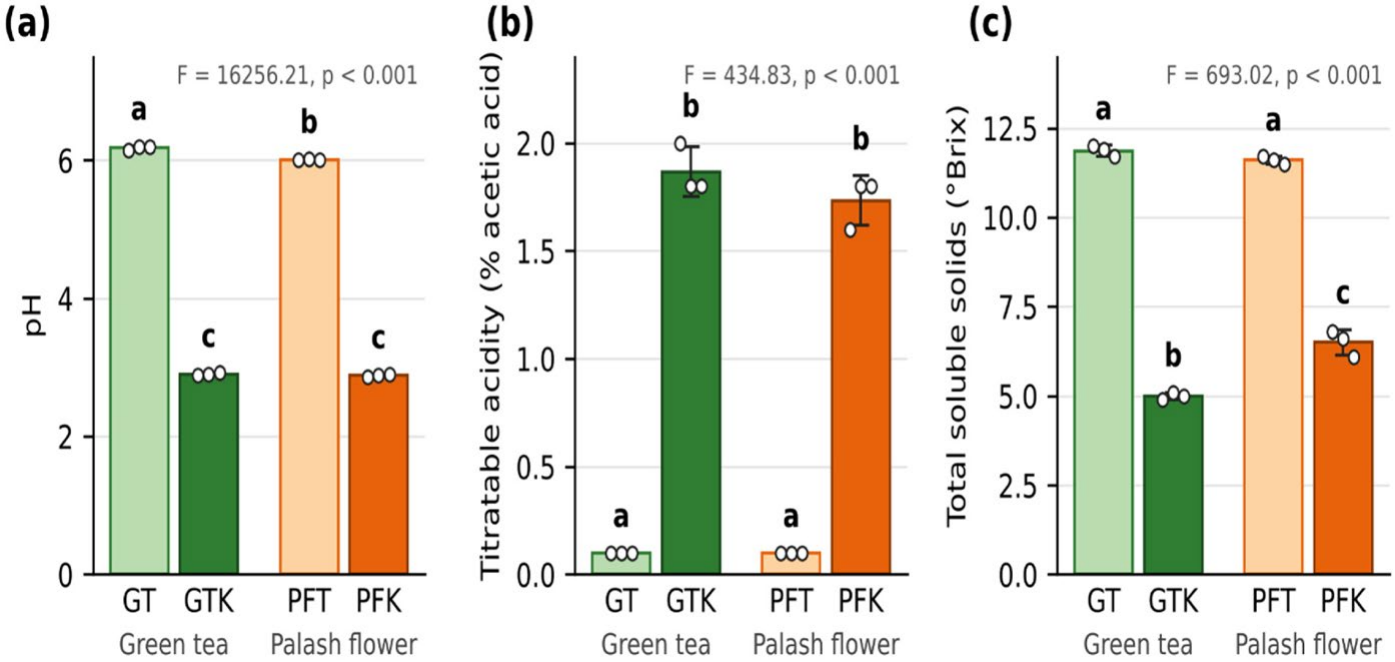
Physicochemical changes in green tea (GT) and *B. monosperma* flower (PFT) substrates before and after 14‐day SCOBY fermentation (GTK and PFK, respectively): (a) pH, (b) titratable acidity (TA, %), (c) total soluble solids (TSS, Brix). Bars represent group means (*n* = 3 independent biological replicates); open circles show individual replicate values. Error bars indicate ± SD. Letters above bars denote Tukey HSD grouping (*p* < 0.05) following one‐way ANOVA; bars sharing a letter are not significantly different. ANOVA: PH, *F* = 16256.21, *p* < 0.001; TA, *F* = 434.83, *p* < 0.001; TSS, *F* = 693.02, *p* < 0.001.

### 3.4 Antioxidant Activity, TPC, and TFC Analysis

Antioxidant activity, TPC, and TFC of the green tea and *B. monosperma* flower substrates before and after SCOBY fermentation are presented in Table 2. Fermentation significantly increased these parameters in both substrates (*p* < 0.05).

**TABLE 2:** Antioxidant activity (DPPH), total phenolic content (TPC), and total flavonoid content (TFC) of unfermented and fermented green tea and palash flower samples.

Treatment	DPPH (%)	TPC (mg GAE/L)	TFC (mg QE/L)
GT	73.09 ± 1.70^c^	236.30 ± 1.00^c^	14.36 ± 1.71^a^
GTK	89.71 ± 1.51^a^	340.16 ± 0.46^a^	85.10 ± 1.73^b^
PFT	60.93 ± 0.71^d^	128.99 ± 0.87^d^	17.18 ± 0.55^a^
PFK	80.41 ± 1.80^b^	263.24 ± 2.92^b^	94.74 ± 1.51^c^

*Note:* Values are mean ± SD (*n* = 3 biological replicates per treatment; 4 treatments × 3 replicates = 12 total measurements). Different superscript letters within a column indicate a significant difference between treatments (two‐way ANOVA with Tukey's HSD post hoc test, *p* < 0.05); columns without superscript letters had a nonsignificant substrate × fermentation interaction (DPPH inhibition: *F* (1,6) = 3.21, *p* = 0.123) and are interpreted via the significant main effects of substrate and fermentation described in the text rather than by all‐pairwise post hoc comparison.

Abbreviations: DPPH, 2,2‐diphenyl‐1‐picrylhydrazyl; GT, green tea (unfermented); GTK, green tea kombucha (fermented); PFK, Palash flower kombucha (fermented); PFT, *B. monosperma* (Palash) flower tea (unfermented); TFC, total flavonoid content, quercetin equivalents; TPC, total phenolic content, gallic acid equivalents.

Before fermentation, GT exhibited higher DPPH radical‐scavenging activity (73.09% ± 1.70%) than PFT (60.93% ± 0.71%). Following 14 days of fermentation, DPPH activity increased to 89.71% ± 1.51% in GTK and 80.41% ± 1.80% in PFK. This increase is consistent with previous reports of enhanced antioxidant potential in kombucha and may result from microbial transformation and increased extractability of bioactive constituents (Chakravorty et al. 2016; Jayabalan et al. 2014).

TPC also increased after fermentation: from 236.30 ± 1.00 to 340.16 ± 0.46 mg GAE/L in GT and from 128.99 ± 0.87 to 263.24 ± 2.92 mg GAE/L in PFT. This increase may be associated with microbial transformation of phenolic constituents, including hydrolysis of conjugated forms and increased release or extractability of phenolic compounds (Jayabalan et al. 2014; Chakravorty et al. 2016; Villarreal‐Soto et al. 2018; Bharti et al. 2026). Although GTK had the highest absolute TPC, PFK showed a greater relative increase over its unfermented substrate.

TFC increased markedly after fermentation, particularly in PFK (from 17.18 ± 0.55 to 94.74 ± 1.51 mg QE/L), which had the highest TFC among the treatments. This increase may be associated with microbial transformation of flavonoid conjugates and enhanced extractability of flavonoid‐related compounds (Villarreal‐Soto et al. 2018; Bharti et al. 2026). The relatively high TFC of PFK may also reflect the flavonoid‐rich composition reported for *B. monosperma* flowers (Kumari et al. 2022).

Two‐way ANOVA indicated significant effects of substrate and/or fermentation status on DPPH activity, TPC, and TFC (*p* < 0.05), and post hoc pairwise comparisons showed significant differences among treatments where applicable. Fermentation therefore altered the antioxidant and phytochemical characteristics of both substrates, although the magnitude of change differed between green tea and *B. monosperma* flower tea.

TPC and DPPH radical‐scavenging activity were strongly and positively correlated across all 12 individual measurements (*r* = 0.9855, *R* ^2^ = 0.9712, *p* < 0.001; Figure 3). This correlation should be interpreted cautiously. Only three biological replicates were available per treatment, and the four treatment groups were well separated in both TPC and DPPH values. The correlation calculated from the four treatment means was similarly high (*r* = 0.992, *n* = 4), indicating that the pooled association was driven largely by differences among treatment groups rather than by a consistent relationship at the biological‐replicate level.

**FIGURE 3:**
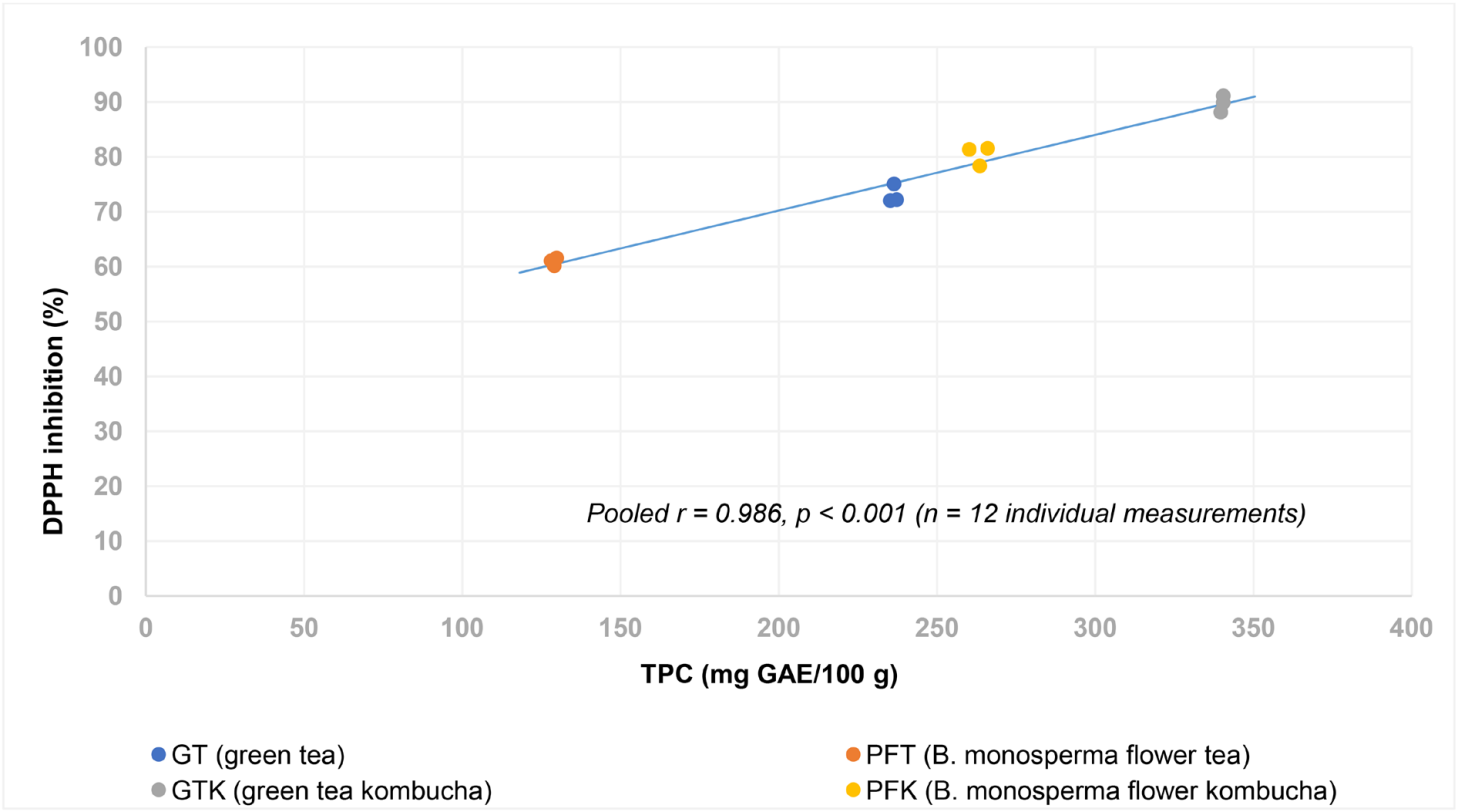
Relationship between total phenolic content (TPC) and DPPH radical‐scavenging activity across 12 individual observations from green tea and *B. monosperma* flower tea before and after fermentation. The fitted linear regression line and Pearson correlation coefficient (*r* = 0.9855) are shown.

Thus, although TPC and DPPH activity increased concordantly across treatments, the correlation alone does not establish that phenolic compounds were the primary contributors to antioxidant activity. In addition, the Folin–Ciocalteu assay is not specific to phenolic compounds and may also respond to other reducing substances in fermented matrices, such as ascorbic acid and reducing sugars. TPC values should therefore be interpreted as an assay‐based estimate of phenolic‐associated reducing capacity rather than a measurement of individual phenolic compounds.

### 3.5 NMR Analysis

^1^H NMR spectra of the tea infusions (D_2_O, 500 MHz) showed marked compositional differences between the unfermented samples (GT, PFT) and their fermented kombucha counterparts (GTK, PFK) (Figure 4, S1 and Table 3a). Spectra from the three biological replicates overlapped closely in peak position and overall shape, indicating good reproducibility. All four spectra were dominated by a very large resonance at *δ* 4.5–4.8 ppm (GT: *δ* 4.76 ppm, integral 363; PFT: *δ* 4.75 ppm, integral 215; GTK: *δ* 4.67 ppm, integral 3559; PFK: *δ* 4.52 ppm, integral 7651). Because no water suppression was applied and the position of this resonance varied between samples, as is characteristic of the temperature‐ and matrix‐dependent D_2_O solvent peak, it was attributed to residual HDO (Gottlieb 1997) and excluded from metabolite‐level interpretation.

**FIGURE 4:**
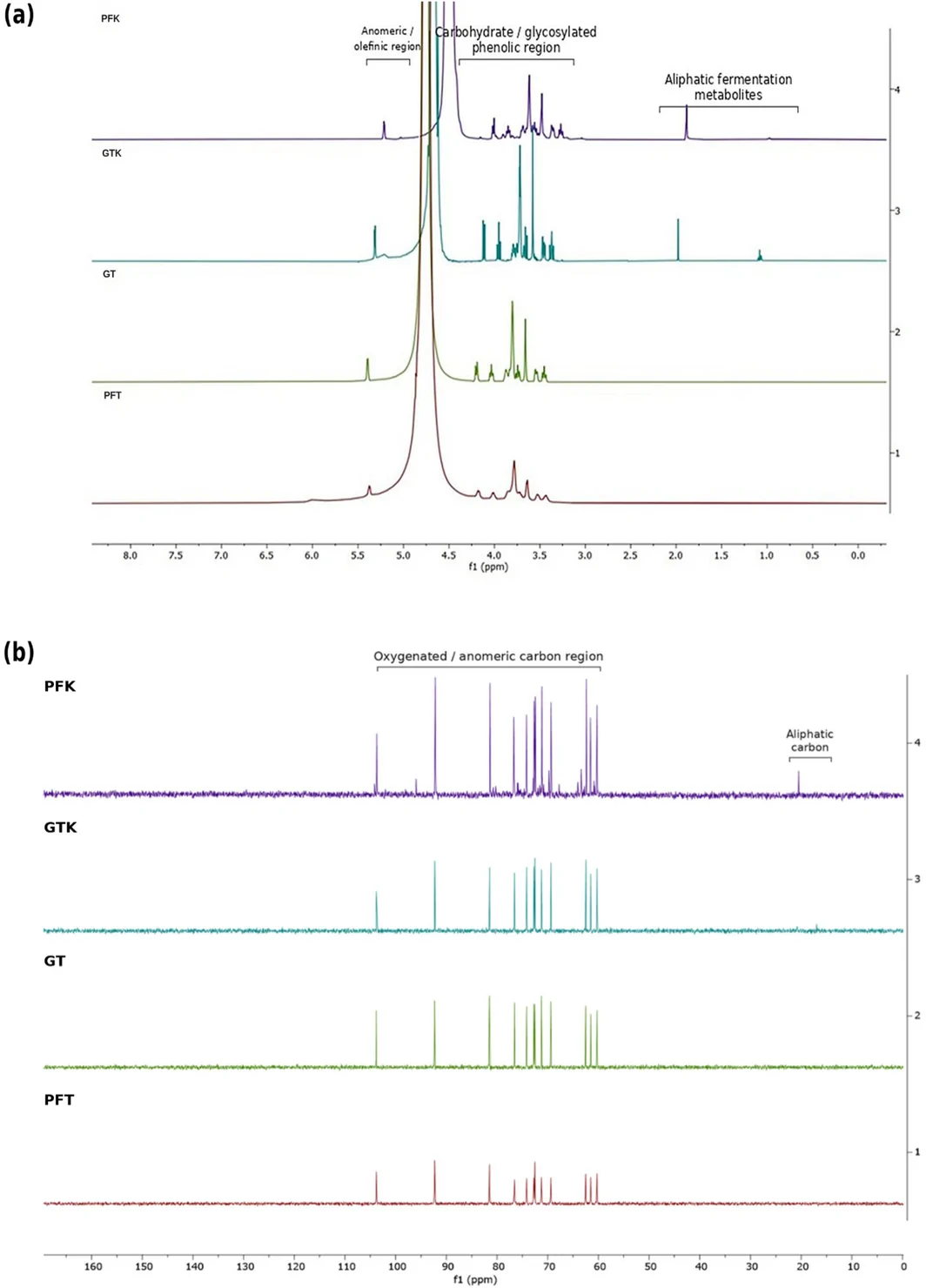
(a) Overlaid ^1^H NMR spectra of *B.monosperma* flower tea (PFT), green tea (GT), green tea kombucha (GTK), and *B. monosperma* flower kombucha (PFK). Bracketed regions indicate broad chemical‐shift regions corresponding to carbohydrate, anomeric/olefinic, and aliphatic fermentation metabolites. (b) Comparative ^13^C NMR spectral profiles of green tea (GT), *B. monosperma* flower tea (PFT), green tea kombucha (GTK), and *B. monosperma* flower kombucha (PFK), highlighting oxygenated/anomeric and aliphatic carbon regions.

**TABLE 3a:** ^1^H NMR chemical shift values of metabolites identified in Green Tea (GT), *B. monosperma* flower tea (PFT), Green tea kombucha (GTK) and *B. monosperma* flower kombucha (PFK).

Peak	Mult.	*δ* range (ppm)	*δ* (ppm)	*J* (Hz)	Relative integral (%)
**Green Tea (GT)**					
A	t	3.41–3.50	3.45	9.50, 9.50	7.08
B	d	3.50–3.57	3.54	10.63	7.44
C	s	3.61–3.68	3.66	—	13.46
D	t	3.69–3.78	3.74	9.56, 9.56	6.30
E	d	3.78–3.90	3.84	35.33	44.19
F	t	3.96–4.06	4.03	8.42, 8.42	7.44
G	d	4.16–4.23	4.20	9.10	7.15
I	s	5.34–5.42	5.40	—	6.94
** *B. monosperma* Flower Tea (PFT)**					
A	m	3.37–3.55	3.47	—	15.72
B	s	3.59–3.66	3.64	—	8.96
C	m	3.68–3.92	3.75	—	47.80
D	q	3.97–4.07	4.02	7.69, 7.69, 8.43	6.45
E	d	4.12–4.23	4.17	7.73	6.60
G	s	5.27–5.43	5.38	—	7.39
H	s	5.81–6.11	5.99	—	7.08
**Green Tea Kombucha (GTK)**					
A	s	1.04–1.12	1.08	—	0.61
B	s	1.95–2.00	1.98	—	0.73
C	s	3.32–3.42	3.37	—	2.96
D	m	3.42–3.49	3.46	—	3.65
E	s	3.52–3.60	3.58	—	9.00
F	t	3.61–3.68	3.66	9.49, 9.49	5.05
G	d	3.69–3.75	3.72	5.23	35.14
H	m	3.75–3.82	3.78	—	6.34
I	t	3.91–3.97	3.95	8.66, 8.66	5.03
J	d	4.08–4.13	4.11	9.18	5.29
L	m	5.15–5.27	5.21	—	20.72
M	d	5.28–5.35	5.31	4.93	5.49
** *B. monosperma* Flower Kombucha (PFK)**					
A	s	0.91–1.01	0.98	—	0.43
B	d	1.81–1.91	1.89	4.68	7.52
C	d	3.15–3.23	3.21	6.73	0.82
D	t	3.24–3.30	3.27	9.34, 9.34	5.08
E	s	3.31–3.39	3.33	—	12.03
F	d	3.43–3.52	3.48	5.32	15.10
G	t	3.53–3.58	3.56	9.89, 9.89	4.27
H	s	3.58–3.66	3.62	—	26.93
I	m	3.66–3.72	3.69	—	5.01
J	t	3.73–3.92	3.85	8.72, 8.72	7.00
K	d	3.94–4.04	4.01	9.32	7.91
L	s	4.11–4.19	4.16	—	0.55
N	s	4.99–5.06	5.03	—	0.65
O	s	5.17–5.26	5.21	—	6.69

*Note:* Relative integral (%) = signal integral expressed as a percentage of the summed integrals of all listed (non‐HDO) resonances within the same spectrum. Values reflect the relative proportion of signal within the resolvable, non‐water region of each spectrum only; they are not normalized to an internal standard and therefore do not represent absolute molar or proton quantities.

In the unfermented teas, the region flanking the solvent peak (*δ* 3.40–4.20 ppm) contained multiple resolved resonances. Based on chemical‐shift comparison only, these are consistent with oxygenated methine and methylene protons typical of carbohydrates and glycosylated phenolics (Tarachiwin et al. 2007; Bartoszek et al. 2018). PFT also showed resonances at *δ* 5.99 and 5.38 ppm, consistent with an aromatic/olefinic ring proton and an anomeric glycosidic proton, respectively. GT showed a broader set of secondary resonances at *δ* 5.40, 4.20, 4.03, 3.84, 3.74, 3.66, 3.54 and 3.45 ppm. The additional GT resonances may reflect the characteristic catechin, caffeine, and theanine content of green tea, whereas the additional PFT signals may reflect the flavonoid glycosides and related phenolics reported in *B. monosperma* flowers. These assignments remain hypotheses for future targeted confirmation, since no standards, spiking, or 2D NMR were used to verify them.

Fermentation introduced additional resonances in both kombucha spectra. GTK showed new signals at *δ* 5.31, 5.21–5.15, 4.11, 3.95, 3.72, 3.58, and 3.37 ppm, together with weak upfield resonances at *δ* 1.98 and 1.08 ppm. PFK showed a broader set of new signals at *δ* 5.21, 5.03, 4.16, 4.01, 3.85, 3.72–3.66, 3.62, 3.56, 3.48, 3.33, 3.27, and 3.21 ppm, together with weak upfield resonances at *δ* 1.89 and 0.98 ppm. Resonances in the *δ* 3.20–4.20 ppm region are tentatively consistent with carbohydrate‐derived protons and other oxygenated metabolites, whereas signals above *δ* 5.00 ppm are consistent with anomeric protons associated with glycosidic compounds (Tarachiwin et al. 2007; Lee et al. 2011). Based on chemical shift alone, the weak upfield resonances at *δ* 0.9–2.0 ppm are consistent with low‐molecular‐weight aliphatic compounds, including short‐chain organic acids that may arise during fermentation. However, the characteristic ethanol coupling pattern (a triplet near *δ* 1.17 ppm coupled to a quartet near *δ* 3.65 ppm) was absent, and no authentic acetic acid standard was used. These signals are therefore conservatively described as an unresolved aliphatic fermentation‐metabolite envelope. Definitive structural assignment would require authentic‐standard spiking and/or two‐dimensional NMR analysis.

The spectral profiles of GTK and PFK differed, but this difference is best explained by the starting materials rather than by differences in fermentation performance. Green tea contributes catechins, caffeine, and amino acids, whereas *B. monosperma* flowers contribute flavonoid glycosides, chalcones, and aurones. The divergent NMR signatures therefore reflect substrate‐specific chemistry combined with microbial transformation. Similar substrate‐dependent differences have been reported between green tea kombucha and herbal or floral variants (Paludo 2017; Aji et al. 2024).

^13^C NMR spectra (125.76 MHz) were broadly consistent with the ^1^H data (Figure 4b and Figure S2). GT and PFT both showed resonances between *δ* 60 and 104 ppm, the range generally reported for oxygenated and anomeric carbons of carbohydrates and glycosylated phenolics (Davis et al. 1996; Valcic et al. 1999). Closely matching peaks near *δ* 103.8, 92.3, 81.5, 76.6, 74.2, 72.7, 71.2, 69.4, and 60–62 ppm indicated a shared carbohydrate‐region carbon framework before fermentation. After fermentation, both kombucha samples retained these carbohydrate‐region resonances, while additional aliphatic carbon signals were observed at *δ* 16.93 ppm in GTK and *δ* 20.49 ppm in PFK, with the latter occurring further downfield than the former. The newly detected signals were cautiously attributed to aliphatic chemical environments on the basis of chemical‐shift positions. However, in the absence of authentic standard confirmation or additional elucidation experiments for structures, these signals could not be explicitly assigned to specific molecular structures or compounds. PFK also showed several resonances in the *δ* 60–81 ppm range (Table 3b), suggesting additional oxygenated carbon environments. These signals could not be assigned to specific metabolites with the available data and would require complementary spectroscopic or chromatographic analyses for definitive identification. These NMR observations are broadly consistent with our earlier HRMS findings, which reported several fermentation‐associated metabolites in *B. monosperma* kombucha, including homobutein, homobutein‐4‐glucoside, quercetin‐3β‐D‐glucoside, epigallocatechin derivatives, gallic acid, and kaempferol (Bharti et al. 2026). The NMR findings are interpreted as complementary evidence of compositional changes associated with fermentation. Given the tentative nature of the spectral assignments and the absence of additional structural confirmation, these findings are used to support the overall compositional interpretation rather than to independently confirm the identities of compounds tentatively annotated in the HRMS analysis. The decrease in pH and TSS and the increase in TPC, TFC, and antioxidant activity remain the more direct and quantitative evidence of fermentation‐associated compositional change.

**TABLE 3b:** ^13^C NMR chemical shift data of Green Tea (GT), *B. monosperma* flower tea (PFT), Green tea kombucha (GTK) and *B. monosperma* flower kombucha (PFK).

Peak	Mult.	*δ* range (ppm)	*δ* (ppm)
**Green Tea (GT)**			
A	s	103.65–103.98	103.82
B	s	92.14–92.50	92.32
C	s	81.38–81.65	81.52
D	s	76.40–76.74	76.57
E	s	73.92–74.38	74.15
F	s	72.54–72.94	72.74
G	s	72.39–72.71	72.55
H	s	71.08–71.39	71.23
I	s	69.22–69.54	69.38
J	s	62.34–62.70	62.52
K	s	61.32–61.69	61.50
L	s	60.07–60.47	60.27
** *B. monosperma* Flower Tea (PFT)**			
A	s	103.57–104.04	103.81
B	s	92.07–92.54	92.30
C	s	81.24–81.77	81.50
D	s	76.09–77.07	76.58
E	s	73.75–74.57	74.16
F	s	72.32–73.19	72.76
G	s	72.29–72.79	72.54
H	s	70.86–71.60	71.23
I	s	68.99–69.78	69.38
J	s	62.20–62.85	62.52
K	s	61.19–61.84	61.51
L	s	59.91–60.66	60.28
**Green Tea Kombucha (GTK)**			
A	s	103.64–103.94	103.79
B	s	92.15–92.41	92.28
C	s	81.32–81.64	81.48
D	s	76.41–76.78	76.59
E	s	73.97–74.33	74.15
F	s	72.61–72.87	72.71
G	s	72.38–72.61	72.52
H	s	71.00–71.40	71.20
I	s	69.22–69.52	69.37
J	s	62.33–62.63	62.48
K	s	61.33–61.70	61.52
L	s	60.09–60.45	60.27
M	s	16.81–17.04	16.93
** *B. monosperma* Flower Kombucha (PFK)**			
A	s	103.96–104.38	104.17
B	s	103.53–103.89	103.71
C	s	95.77–96.16	95.97
D	s	92.01–92.36	92.18
E	s	81.23–81.56	81.40
F	s	80.54–80.95	80.74
G	s	80.03–80.50	80.27
H	s	76.45–76.93	76.69
I	s	75.84–76.11	75.98
J	s	73.93–74.41	74.17
K	s	72.63–73.10	72.86
L	s	72.56–72.88	72.68
M	s	72.26–72.56	72.45
N	s	71.39–71.77	71.58
O	s	70.97–71.30	71.14
P	s	70.67–70.94	70.81
Q	s	69.60–69.94	69.77
R	s	69.54–69.94	69.72
S	s	69.13–69.54	69.36
T	s	69.08–69.39	69.24
U	s	67.52–67.98	67.75
V	s	63.94–64.18	64.06
W	s	63.20–63.59	63.40
X	s	62.21–62.57	62.39
Y	s	61.30–61.85	61.58
Z	s	60.66–61.08	60.87
A1	s	60.06–60.50	60.28
B1	s	20.32–20.66	20.49

*Note:* δ, chemical shift (ppm); Mult., multiplicity (s = singlet). The δ range represents the range of chemical shifts observed across replicate spectra, while δ (ppm) indicates the representative chemical shift. Peak labels identify individual resonances.

## 4 Conclusion

This study characterized the microbial and metabolic changes during the fermentation of *B. monosperma* flower kombucha by combining live‐cell imaging, semi‐quantitative ImageJ analysis, and ^1^H/^13^C NMR spectroscopy. Fermentation significantly improved the physicochemical properties and bioactive potential of both green tea and *B. monosperma* flower substrates, as shown by changes in pH, total soluble solids, titratable acidity, total phenolic content, total flavonoid content, and antioxidant activity. Live‐cell imaging and ImageJ analysis showed progressive SCOBY development and increasing structural organization during fermentation, while NMR analysis revealed fermentation‐associated changes in the chemical composition of the beverages. Collectively, these findings highlight the potential of *B. monosperma* flower kombucha as a functional fermented beverage and provide a scientific basis for its further development and valorization.

## Author Contributions

**Suman Bharti:** investigation, writing – original draft. **Dinesh Chandra Rai:** conceptualization, validation, supervision. **Arvind Kumar:** investigation, supervision, writing – review and editing. **Anand Kumar Pandey:** data curation, formal analysis. **Yada Nolvachai:** funding acquisition, resources, writing – review and editing. **Dipendra Kumar Mahato:** resources, funding acquisition, writing – review and editing.

## Funding

The authors have nothing to report.

## Conflicts of Interest

The authors declare no conflicts of interest.

## Supporting information


**Figure S1:** ^1^H NMR spectra of three biological replicates of green tea and *Butea monosperma* (Palash) flower kombucha samples. (A) Biological replicate 1, (B) biological replicate 2, and (C) biological replicate 3. GT, green tea infusion; GTK, green tea kombucha; PFT, Palash flower tea infusion; PFK, Palash flower kombucha. Spectra are vertically offset for clarity. The annotated regions indicate broad spectral regions corresponding to anomeric/olefinic, carbohydrate/glycosylated/phenolic, and aliphatic fermentation‐associated resonances.
**Figure S2:** ^13^C NMR spectra of three biological replicates of green tea and *B. monosperma* (Palash) flower kombucha samples. (A) Biological replicate 1, (B) biological replicate 2, and (C) biological replicate 3. GT, green tea infusion; GTK, green tea kombucha; PFT, Palash flower tea infusion; PFK, Palash flower kombucha. Spectra are vertically offset for clarity. The annotated regions indicate oxygenated/anomeric carbon and aliphatic carbon environments.

## Data Availability

The data that support the findings of this study are available on request from the corresponding author. The data are not publicly available due to privacy or ethical restrictions.
