# Supplementary material for: Deciphering Butea monosperma Kombucha Fermentation Through NMR and Live‐Cell Imaging
Source: Food Sci Nutr. 2026 Nov 3;14(11):e72531. doi: 10.1002/fsn3.72531 (PMC13731839; doi:10.1002/fsn3.72531)
Supplement: Supplementary file 1 [file FSN3-14-e72531-s001.docx]

**Figure S1.** ¹H NMR spectra of three biological replicates of green tea and *B. monosperma* (Palash) flower kombucha samples. (A) Biological replicate 1, (B) biological replicate 2, and (C) biological replicate 3. GT, green tea infusion; GTK, green tea kombucha; PFT, Palash flower tea infusion; PFK, Palash flower kombucha. Spectra are vertically offset for clarity. The annotated regions indicate broad spectral regions corresponding to anomeric/olefinic, carbohydrate/glycosylated/phenolic, and aliphatic fermentation-associated resonances.

**Figure S2.** ¹³C NMR spectra of three biological replicates of green tea and *B. monosperma* (Palash) flower kombucha samples. (A) Biological replicate 1, (B) biological replicate 2, and (C) biological replicate 3. GT, green tea infusion; GTK, green tea kombucha; PFT, Palash flower tea infusion; PFK, Palash flower kombucha. Spectra are vertically offset for clarity. The annotated regions indicate oxygenated/anomeric carbon and aliphatic carbon environments.
